# Reliability of medical students' vaccination histories for immunisable diseases

**DOI:** 10.1186/1471-2458-8-121

**Published:** 2008-04-15

**Authors:** Sabine Wicker, Regina Allwinn, René Gottschalk, Holger F Rabenau

**Affiliations:** 1 Occupational Health Service, Hospital of the Johann Wolfgang Goethe University of Frankfurt, Theodor-Stern-Kai 7, 60590 Frankfurt, Germany; 2 Institute of Medical Virology, Hospital of the Johann Wolfgang Goethe University of Frankfurt, Paul-Ehrlich-Str. 40, 60596 Frankfurt, Germany

## Abstract

**Background:**

Medical students come into contact with infectious diseases early on their career. Immunity against vaccine-preventable diseases is therefore vital for both medical students and the patients with whom they come into contact.

**Methods:**

The purpose of this study was to compare the medical history and serological status of selected vaccine-preventable diseases of medical students in Germany.

**Results:**

The overall correlation between self-reported medical history statements and serological findings among the 150 students studied was 86.7 %, 66.7 %, 78 % and 93.3 % for measles, mumps, rubella and varicella, conditional on sufficient immunity being achieved after one vaccination. Although 81.2 % of the students' medical history data correlated with serological findings, significant gaps in immunity were found.

**Conclusion:**

Our findings indicate that medical history alone is not a reliable screening tool for immunity against the vaccine-preventable diseases studied.

## Background

Medical students are often exposed to infectious diseases in the course of their clinical training.

It is therefore important to identify and limit any possible gaps in immunity of vaccine-preventable diseases before initial patient contact [1,2]. Reports by Breuer [3] confirm that health care workers (HCWs) and medical students are also a source of infection for patients. HCWs and their employers should therefore be responsible for protecting health care personnel and patients against hospital infections [4-7].

The Standing Committee on Vaccination (STIKO) at the German Robert Koch-Institute (RKI) has recommended two doses of MMR vaccine to all children to prevent outbreaks of measles, mumps and rubella. The first dose should be given at 11–14 months, the second vaccination at 15–23 months. In 2006, the STIKO issued a recommendation for one dose of varicella vaccine to be given universally to children at 11–14 month. HCWs without evidence of immunity should have one dose of MMR and two doses of varicella vaccination.

It is in the interests of the medical students to know their own immune status against the immunisable viral infections, but the individual student cannot be forced to undergo vaccination. However, faculty has to bear the costs of required vaccinations.

The purpose of this study was to compare the medical history with the serological status of selected vaccine-preventable viral diseases (measles, mumps, rubella and varicella) of medical students in their first clinical semester at the Johann Wolfgang Goethe-University in Frankfurt, Germany. The aim was to determine whether a serological examination of the immune status was required before vaccination or if a self-reported medical history report alone was sufficient. The main focus was on epidemiological and economical factors.

## Methods

### Study Group

At the Johann Wolfgang Goethe-University, 270 medical students (172 [63.7 %] females; 98 [36.3 %] males) were enrolled in their first clinical semester in October 2005. The students were informed about the study in a lecture. Overall, 150 (55.6 %) students (110 [73.3 %] females; 40 [26.7 %] males) completed the standardised questionnaire and took part in the study. Individuals with unknown histories were not included in the study group. The median age was 23.4 years (range 20 – 45 years).

Data were obtained by a voluntary questionnaire. Before the study was carried out, the head of the *Ethics Committee of the University hospital Frankfurt *confirmed, that there was no special ethical approval required, because the evaluation of our study data was totally anonymous and there were no additional blood samples necessary. We can confirm that participants cannot be identified from the material presented and that no plausible harms to participating individuals arise from the study.

### Serological Tests

Serological tests were carried out for varicella zoster virus (VZV), measles, mumps and rubella. Rubella specific IgG-antibodies were tested by means of the Rubella IgG-MEIA-assay (AxSYM system, Abbott Diagnostics Divison, Wiesbaden, Germany). Anti-measles virus-IgG, anti-mumps-IgG, and anti-VZV-IgG were determined by means of Enzygnost^® ^Anti-Measles Virus-IgG, Enzygnost^® ^Anti-Parotis Virus-IgG, or Enzygnost^® ^Anti-VZV-IgG (all from Dade Behring, Marburg, Germany) respectively in the Behring ELISA processor BEP 2000. Test results were recorded as positive, borderline or negative, as outlined by the manufacturers. The sensitivity and specificity of the tests were detailed by the manufactures as follows: 98.0 %, 99.0 % (Rubella IgG-MEIA); 99.6 %, 100.0 % (Enzygnost^® ^Anti-Measles Virus-IgG); 95.4 %, 93.7 % (Enzygnost^® ^Anti-Parotis Virus-IgG); and 99.3 %, 100.0 % (Enzygnost^® ^Anti-VZV-IgG).

As regards measles, mumps and VZV, clear positive antibody results (quantified in international units per litre [IU/ml]) indicate a humoral immunity; borderline serological test results show an insufficient humoral immunity and consequential, seronegative indicate no detectable humoral immunity. Students with borderline or negative serological results were assessed as "non-immune" and were vaccinated.

Rubella-specific antibody measurement was carried out with an ELISA. In the case of low-level immunity, a hemagglutination inhibition assay (HI) was also performed. HI-titres of 1: ≥ 32 or ELISA values ≥ 35 IU/l indicate sufficient rubella immunity, low level immunity was defined by a titre of 1:16 (HI) or ELISA values between 20–35 IU/l. HI-titres ≤ 1:8 or ELISA values of 1:<20 IU/l were assessed as "non-immune".

### Medical history data

**Figure 1:**
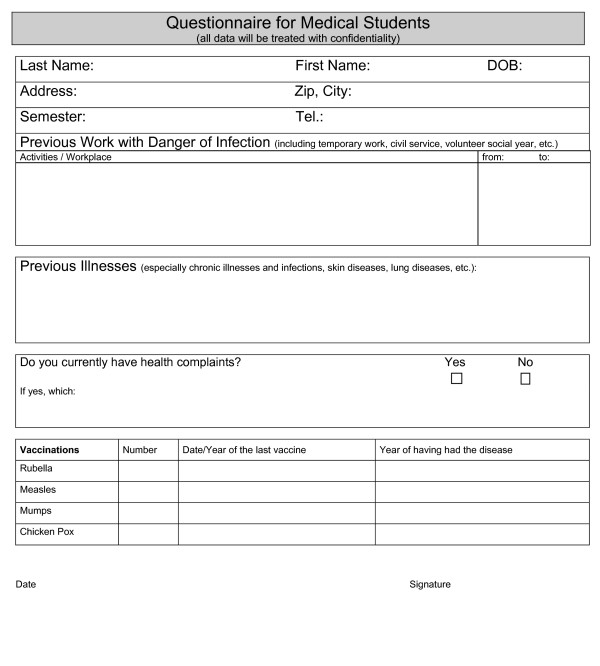
Questionnaire for medical students.

The students recorded their vaccination status (date and total number of vaccinations) against measles, mumps, rubella and varicella by means of a standardised questionnaire (Fig. 1). Self-reported medical history data regarding infection with the relevant viruses was also documented. The reliability of the self-reported medical history statements was compared with the serological findings. For purposes of these analyses, students claiming "don't know" for history of disease or vaccination were excluded, only documentary evidence was accepted for a history of vaccination.

### Correlation factor

The overall correlation (in %) of the medical history with the serological results was evaluated as followed:

Either

- sufficient immunity was achieved by a single vaccination, or

- insufficient immunity followed a single vaccination. In this case, borderline and negative serological results were recorded as non-immune and compatible with the medical history, while positive results were recorded as incompatible with the medical history.

### Predictive value

*Positive predictive value (PPV): *The reliability of a positive history of disease or vaccination in predicting immunity.

*Negative predictive value (NPV): *The reliability of a negative self-reported history of disease or vaccination in predicting susceptibility.

## Results

The overall correlation between medical history statements and serological findings among the 150 students studied was 86.7 %, 66.7 %, 78 % and 93.3 % for measles, mumps, rubella and varicella, provided that sufficient immunity was achieved after a single vaccination.

**Figure 2:**
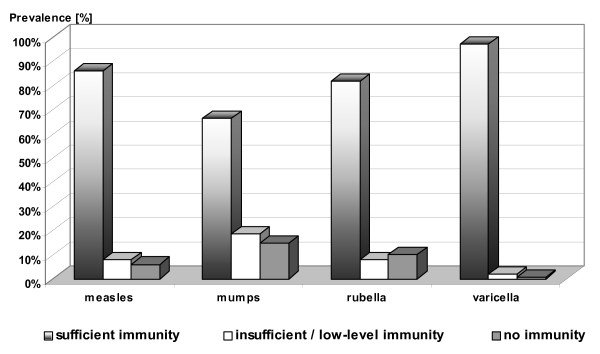
Measles, mumps, rubella and VZV-seroprevalence (n = 150).

Measles-specific antibodies could be identified in 94 % of the students (n = 141), with 86 % (n = 129) achieving sufficient immunity, 8 % (n = 12) with insufficient immunity (measles virus IgG borderline values) and 6 % (n = 9) with no immunity (Fig. 2). The medical history statements showed that 46 % of the students (n = 69) had had either at least two measles vaccinations (n = 62) or measles itself (n = 7). About 14.5 % (n = 10) of them showed either an insufficient or total lack of immunity serologically. Among the seven students who reported having had neither measles nor a vaccination against it, three were sufficiently immune in the serological test (Tab. 1). Probability estimates are presented in Table 2.

**Table 1:** Correlation of the medical history of vaccination or virus-specific disease and serological results (n = 150)

	**measles**				**mumps**			
		
		serological results				serological results		
	no. of students	positive	borderline	negative	no. of students	positive	borderline	negative
History of disease	7	**5**	**0**	**2**	8	**7**	**0**	**1**
History of at least 2 vaccinations	62	**54**	**7**	**1**	56	**46**	**7**	**3**
History of 1 vaccination	74	**67**	**5**	**2**	76	**42**	**21**	**13**
No history of disease or vaccination	7	**3**	**0**	**4**	10	**4**	**1**	**5**
Overall correlation (in %) of serological results and medical history data *(conditional on sufficient immunity being achieved after a single vaccination)*	150	86.7 %			150	66.7 %		
Overall correlation (in %) of serological results and medical history data *(insufficient immunity following one vaccination *^1^)	150	46.7 %			150	61.3 %		

	**rubella**				**varicella**			
		
		serological results				serological results		
	no. of students	positive	borderline	negative	no. of students	positive	borderline	negative

History of disease	17	**15**	**0**	**2**	144	**140**	**3**	**1**
History of at least 2 vaccinations	51	**39**	**12**	**0**	0	**0**	**0**	**0**
History of 1 vaccination	72	**61**	**4**	**7**	0	**0**	**0**	**0**
No history of disease or vaccination	10	**8**	**0**	**2**	6	**6**	**0**	**0**
Overall correlation (in %) of serological results and medical history data *(conditional on sufficient immunity being achieved after a single vaccination)*	150	78 %			150	93.3 %		
Overall correlation (in %) of serological results and medical history data *(insufficient immunity following one vaccination *^1^)	150	44.7 %			150	93.3 %		

^1^*borderline and negative results are assessed as correlating to the medical history data*

**Table 2:** Positive predictive value (PPV) of self-reported History of Disease (HD) or Vaccination against Disease (HV) in predicting immunity (n = 150). HV one: one vaccination, HV two: two vaccinations

	PPV
Measles	
HD	71.4%
HV one	97.3%
HV two	98.4%
Mumps	
HD	87.5%
HV one	82.9%
HV two	94.6%
Rubella	
HD	88.2%
HV one	88.5%
HV two	100.0%
Varicella	
HD only	99.3%

Mumps-specific antibodies were detected in 85.3 % (n = 128) of the students. In detail: 66 % (n = 99) had sufficient immunity, 19.3 % (n = 29) had insufficient immunity (mumps virus IgG borderline values) and 14.7 % (n = 22) had no immunity (Fig. 2). The correlation with the data from the questionnaire showed that 17.2 % (n = 11) of the students who reported having had either at least two mumps vaccinations (n = 56) or mumps itself (n = 8) had serologically an insufficient immunity or none at all. Among the 10 students who reported having had neither mumps nor a mumps vaccination, four showed a sufficient immunity (Tab. 1). The overall correlation between the serological and medical history data for mumps immunity was 66.7 %, provided that sufficient immunity was achieved after a single vaccination.

Rubella-specific antibodies could be found in 92.7 % of the students (n = 139), with 82 % (n = 123) having sufficient immunity (IgG ≥ 35 IU/l), 10.7 % (n = 16) having low-level immunity (IgG ≥ 20 - <35 IU/l), and 7.3 % (n = 11) completely non-immune (Fig. 2). Insufficient or a total lack of immunity was shown in 20.6 % (n = 14) of the students who reported either at least two rubella vaccinations (n = 51) or having had the german measles itself (n = 17). Among the 10 students who reported having had neither rubella nor a vaccination against it, eight students showed sufficient immunity (Tab. 1).

With regards to varicella serology, 99.3 % (n = 149) of the students showed specific antibodies. In detail: 97.3 % (n = 146) of the students showed sufficient varicella immunity, 2 % (n = 3) had an insufficient immunity (borderline values: 100 – 150 IU/l) and one student (0.7 %) was seronegative (Fig. 2). Based on self-reported medical history data, 144 students (96 %) reported having had chicken pox in their childhood, none had a history of a VZV vaccination, four (2.6 %) showed an insufficient or lack of immunity serologically. Six students (4 %) who could not remember having had chicken pox showed sufficient VZV immunity serologically. To summarise, the self-reported medical history statements of 140 (93.3 %) students correlated with the serological findings (Tab. 1). Self-reported history of varicella had a high PPV (99.3%) for a test result positive for anti-varicella virus antibodies (Tab. 2).

Overall, 81.2 % of the students' medical history data correlated with serological results, thus significant gaps in immunity were found.

## Discussion

Medical students come into contact with infectious diseases early on their career [4,5].

The aim of our study was to determine whether the data of a standardised questionnaire about the medical history correlated with serological results. In the event of a high correlation rate the necessity of vaccinations would be demonstrated by the questionnaire alone, thereby avoiding cost-intensive serological analysis. In view of limited financial budgets in the public health system, economical aspects – such as the avoidance of serological analysis or vaccinations – should be given serious consideration [8,9].

The serological results of our study showed that 14 % of the students had insufficient measles immunity or were totally non-immune. For mumps, rubella and chickenpox, 34 %, 18 % and 2.7 % had insufficient or no immunity. The serological data in this study is similar to data from a non-student study of a comparable age group (20 – 29 years) from the University Hospital of Frankfurt [10] as well as to results from studies on medical students at the University of Frankfurt [11] and Basel [2].

With regards to the sensitivity of the assays used, it should be taken into account that false negative ELISA results might distort the actual rate of humoral immunity [12]. These assays are nevertheless commonly used as screening tests to determine immunity status because they are less labor- and time-intensive than cell culture-based neutralisation assays which detect neutralising antibodies. International antibody standards exist for measles and rubella but not for mumps. Compared with neutralisation assays, up to 30 % of ELISA false negative mumps antibody results were recorded [12]. This is often due to the relatively high serum-dilution factors used in ELISA tests.

Nevertheless, it is clear from the study that it is vital to assure total immunity before initial patient contact. This is important not only for the students' individual protection but also for the prevention of hospital infections [13-16].

From an economical point of view three different vaccination strategies are possible:

1. Serological testing of all students, elimination of individual gaps in immunity.

2. Recording medical students' past viral diseases or vaccinations, elimination of lapses in immunity.

3. Vaccination of all students without prior serological testing or reports (In this case, it should be taken into consideration that the administration of an attenuated live vaccine to a large patient population may result in side effects and thereby reduce the acceptance rate of vaccinations).

The conformity of the results of the questionnaire with the serological data was moderate to unsatisfactory. For measles, mumps, rubella and VZV the total correlation rate was 86.7 %, 66.7 %, 78 % and 93.3 %, provided that sufficient immunity was achieved after a single vaccination. This assumption is backed up by data from the WHO which indicate that 85 – 90 % of people who receive a single dose of the measles vaccine show a sufficient immunity [17]. For rubella, mumps and VZV, a single dose vaccine achieves immunity of ≥ 95 %, 75–99 % and 95 % of the recipients [18-20].

The present study shows that 14.5 % of the students who reported having had two measles vaccinations or measles itself showed insufficient or no immunity serologically. This was also valid for 17.2 % of the students regarding mumps. In the case of the mumps ELISA, the limited sensitivity of the assay should be taken into consideration [12]. Assuming that borderline ELISA results are likely representing immunity, instead of 17.2 %, only 6.3 % of those students would show no immunity. Additionally, the overall correlation between the serological results and the medical history data would increase to 85.3 %.

In the case of rubella and VZV, 20.6 % and 2.8 % of the students who reported having had at least two rubella vaccinations, rubella itself or chickenpox did not show a sufficient immunity serologically. If the students' self-reported medical history data is correlated with the serological results, it should be taken into account that the reliability of information based on history about the specific aetiology of a disease might limited [21]. The fact that different parameters such as test sensitivity or reliability of information based on history might influence the correlation should certainly be taken into account.

Overall, self-reported history of varicella and measles had a higher PPV (99.3%; 96.5% respectively) than self-reported history of rubella (93.6%) and mumps (87.8). In addition, self-reported history of vaccinations against measles, mumps and rubella had a higher PPV than self-reported history of these diseases and two vaccinations had a higher PPV than one vaccination.

Similar data of PPV were published in several other studies, which showed that reliability upon historical information alone is inadequate to identify all susceptible individuals [22-24]. For this reason, it was decided, at Frankfurt university hospital, to continue the serological testing of all medical students. Based on the test results, individual gaps in immunity can be closed with vaccination.

However, among HCWs at occupational risk of viral immunisable diseases, the need for vaccination can be reduced by combining historical and serological screening [25]. Nevertheless, using only data based on history as a criterion of immunity to immunisable viral diseases, would not achieve the goal of reducing susceptibility to zero in the medical student population [23].

## Conclusion

Our study indicates that a students' medical history alone is not a reliable screen for determining immune status for the vaccine-preventable diseases studied.

## Competing interests

The author(s) declare that they have no competing interests.

Herewith we confirm that there are no potential conflicts of interests and any sources of funding.

## Authors' contributions

SW designed the study together with HFR. SW: author of the publication, data analysis, study design. RA: scientific supervision. RG: statistical analysis. HFR: scientific supervision, study design, co author of the publication. Finally, all authors participated, read and approved the final manuscript.

## Pre-publication history

The pre-publication history for this paper can be accessed here:


